# Targeting CCN2 protects against progressive non-alcoholic steatohepatitis in a preclinical model induced by high-fat feeding and type 2 diabetes

**DOI:** 10.1007/s12079-022-00667-1

**Published:** 2022-01-17

**Authors:** Jing Ren, Xiaoyu Wang, Sarah N. Parry, Christine Yee, Mark D. Gorrell, Susan V. McLennan, Stephen M. Twigg

**Affiliations:** 1 Greg Brown Diabetes and Endocrinology Research Laboratories, Sydney Medical School (Central), Faculty of Medicine and Health, Charles Perkins Centre, The University of Sydney, Sydney, NSW 2006 Australia; 2 Department of Endocrinology, Royal Prince Alfred Hospital, Camperdown, NSW 2050 Australia; 3 Centenary Institute, The University of Sydney, Camperdown, NSW 2050 Australia; 4 Faculty of Medicine and Health, The University of Sydney, Sydney, NSW 2006 Australia; 5 New South Wales Health Pathology, Camperdown, NSW 2050 Australia

**Keywords:** CCN2, NAFLD, Type 2 diabetes, Neutralizing antibody, Inflammation, Fibrosis

## Abstract

Type 2 diabetes is an independent risk factor for non-alcoholic steatohepatitis (NASH) progression and its mediators have not been resolved. In this study, a pathogenic role of cellular communication network factor 2 (CCN2) protein in NASH pathology, was investigated in an established preclinical NASH model. Male wild type C57BL/6 mice received either Chow or high fat diet (HFD) for 26 weeks, with some mice in each group randomly selected to receive low dose streptozotocin (STZ: 3 i.p. injections, 65 mg/kg) at 15 weeks to induce type 2 diabetes. In the final 10 of the 26 weeks mice from each group were administered i.p. either rabbit anti-CCN2 neutralizing antibody (CCN2Ab) or as control normal rabbit IgG, at a dose of 150 µg per mouse twice/week. NASH developed in the HFD plus diabetes (HFD+DM) group. Administration of CCN2Ab significantly downregulated collagen I and collagen III mRNA induction and prevented pro-inflammatory MCP-1 mRNA induction in HFD+DM mice. At the protein level, CCN2Ab significantly attenuated collagen accumulation by PSR stain and collagen I protein induction in HFD+DM. Phosphorylation of the pro-fibrotic ERK signalling pathway in liver in HFD+DM was attenuated by CCN2Ab treatment. Intrahepatic CCN1 mRNA was induced, whereas CCN3 was downregulated at both the mRNA and protein levels in HFD+DM. CCN3 down-regulation was prevented by CCN2Ab treatment. This in vivo study indicates that CCN2 is a molecular target in NASH with high fat diet and diabetes, and that regulation of ERK signalling is implicated in this process.

## Introduction

Cellular communication network factor 2, CCN2, is a cysteine-rich secreted protein and often a down-stream mediator of transforming growth factor-β (TGF-β). Previously known as connective tissue growth factor (CTGF), the biological roles of CCN2 include the regulation of cell proliferation, differentiation, survival, adhesion, extracellular matrix (ECM) accumulation, and angiogenesis. CCN2 has been regarded as a candidate responsible for fibrotic change (Shi-wen et al. 2006). Upregulation of hepatic CCN2 occurs in fibrotic liver in humans (Paradis et al. 1999) and CCN2 expression in liver parenchyma correlated positively with progressive NASH in a monkey model (Chen et al. 2018). Moreover, inhibition of CCN2 attenuated liver fibrogenesis in a carbon tetrachloride-induced liver fibrosis mouse model (Uchio et al. 2004). CCN2 is implicated in a number of end-organ complications in diabetes, especially in tissues where accumulation of ECM components occurs including the kidney in nephropathy, and the heart in cardiomyopathy (Frazier et al. 1996).

In previous studies published by our laboratory, CCN2 was reported to be upregulated in a clinically relevant murine high fat fed and diabetes NASH experimental model (Lo et al. 2011). Considering its known biological behaviour, CCN2 may not only be a marker, but also a mediator, in fibrosis progression in NASH. In order to investigate whether CCN2 may be involved in the worsening of NASH pathology, systemic administration of anti-CCN2 neutralizing antibody as an intervention was studied in the HFD alone, diabetes alone and the HFD plus diabetes mouse model (Lo et al. 2011), both in terms of effects on NASH with fibrosis and in exploring potential cellular mediators of CCN2 effects.

CCN2 is one of the six CCN family members, as matricellular associated proteins involved in mitosis, adhesion, apoptosis, extracellular matrix production, growth arrest and migration of multiple cell types (Brigstock 2003). CCNs are expressed early in development and are recruited by cells to facilitate different tissue functions, especially in wound healing and several diseases (Yeger and Perbal 2007). Regarding the role of CCN members other than CCN2 in liver disease, CCN1 in a fat fed model has been shown to prevent NAFLD, especially steatosis, to induce apoptosis of hepatic pro-fibrotic activated stellate cells and to reduce the expression of fibrosis markers (Borkham-Kamphorst et al. 2014). CCN3 has been reported to potentially be anti-fibrotic, partly through acting to inhibit pro-fibrotic gene induction as well as through inhibiting CCN2 gene expression (Leask 2009). In contrast, the involvement of CCN4, CCN5 and CCN6 in liver fibrosis is largely unknown. In this study, in addition to the main aim of targeting CCN2 to determine if that approach may prevent NASH fibrosis, we also investigated potential mechanisms of CCN2 effects in NASH liver, including on other CCN members.

## Materials and methods

### Animal and experimental design

**Fig. 1:**
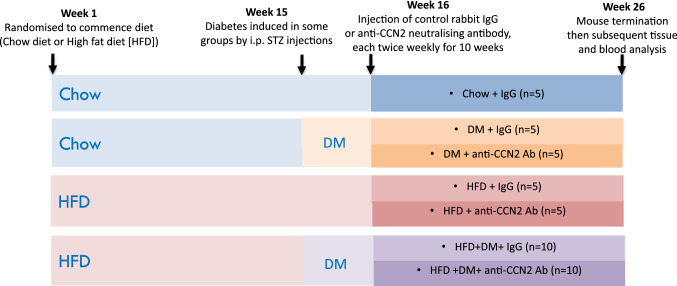
Schematic of the study protocol and time course. This includes the mouse groups examined, number of mice in each group, the diets provided, diabetes induction, then subsequent anti-CCN2 antibody or control IgG administration. For more detail refer to the study Methods

Male C57BL/6 J mice aged 5 weeks, a total of n = 45, were purchased from the Animal Resource Centre (Perth, Western Australia), housed with a 12 h light /dark cycle, and provided food and water ad libitum. After 1 week adaptation, the mice were randomly allocated into 2 groups fed either with standard chow (CHOW; 12% kcal fat content) or a high fat diet (HFD; 45% kcal fat content) each for 26 weeks. The HFD was prepared in-house with a formula based on rodent diet no. D12451 from Research Diets (New Brunswick, NJ), as published by ourselves (Lo et al. 2011), and others (Rossmeisl et al. 2003). To induce type 2 diabetes some mice from each group were randomly selected and injected at 15 weeks with low dose streptozotocin (STZ: 3 × 65 mg/kg i.p.). For the final 10 weeks, from week 16 to week 26, mice randomly selected from the diabetes alone, HFD alone, and HFD plus diabetes (HFD + DM) groups, were injected intraperitoneally with either neutralizing CCN2Ab (150 µg/per mouse, twice per week, diluted in Phosphate Buffered Saline), or normal control rabbit IgG (150 µg/per mouse, twice per week, diluted in Phosphate Buffered Saline). Animals were weighed and blood glucose levels were checked weekly throughout the study (Lo et al. 2011). Insulin tolerance tests (ITT) were performed 1 week before termination, as previously described (Lo et al. 2011). At termination all mice were anesthetized and mice were euthanased by exanguination and several indices were examined in the collected liver tissue. Both HFD and diabetes induction are required in the model especially to induce robust liver fibrosis in the model (Lo et al. 2011). The mouse groups generated and studied, with respective mouse numbers per group, are shown in Schematic Fig. 1. All animal procedures were approved by the animal ethics committee of Sydney South-West Area Health Services, and Sydney Local Health District, NSW, Australia.

### Generation of anti-CCN2 neutralizing antibody

The neutralizing anti-CCN2 polyclonal antibody was commercially generated by IMVS (Adelaide, South Australia). A New Zealand White Rabbit was injected s.c. with 160 µg of full length recombinant human (rh) CCN2 (Wang et al. 2010) in Freund’s adjuvant, followed by four further sequential s.c. injections of the 160 µg full length rhCCN2 at 3 week intervals, also in Freund’s adjuvant. The antiserum was collected as a terminal bleed and was found to detect full-length rhCCN2 by Western blot (not shown). The IgG total amount purified from rabbit serum was 72 mg. The CCN2Ab as 10 µg/mL of the IgG, when applied to murine H9C2 cardiomyocytes, was found to prevent induction of Collagen I, Collagen III, and Fibronectin mRNA levels at 24 h (not shown), otherwise caused by addition of 500 ng/mL of rhCCN2 to unconditioned media (Wang et al. 2010).

### Quantitative real-time polymerase chain reaction

Total RNA was isolated from liver tissue by PureLink RNA mini Kit (Life technologies, USA) according to the manufacturer’s instructions. Total RNA (2000 ng) from each sample was reverse-transcribed to cDNA using Random Hexamer Primer (Invitrogen, CA, USA) and Superscript III reverse transcriptase (Invitrogen). The mRNA levels of MCP-1, TNF-ɑ, IL-1ß, IL-6, Collagen-I, -III, -IV, -VI, FN, CCN2, TGF-ß1, TIMP-1, CCN1 and CCN3, were determined by quantitative real-time PCR using SYBR green fluorophore (Invitrogen). The primer pair sequences used for respective mRNA species are shown in Table 1. In each case, the mRNA of interest expression level was normalized by 18 s ribosomal RNA (Lo et al. 2011). The relative amount of target mRNA in each sample was determined by applying the threshold cycle to the standard curve.

**Table 1:** Primer sequences utilized for qPCR end-points

	Forward	Reverse
MCP-1	AGGTCCCTGTCATGCTTCTG	GCTGCTGGTGATCCTCTTGT
TNF-ɑ	CCCCAAAGGGATGAGAAGTT	CACTTGGTGGTTTGCTACGA
IL-1ß	GACCTTCCAGGATGAGGACA	AGCTCATATGGGTCCGACAG
IL-6	TTCACAAGTCCGGAGAGGAG	TTCTGCAAGTGCATCATCGT
Coll I	CCCCGGGACTCCTGGACTT	GCTCCGACACGCCCTCTCTC
Coll III	CCTGGAGCCCCTGGACTAATAG	GCCCATTGCACCAGGTTCT
Coll IV-ɑ1	ATCCGGCCCTTCATTAGC	ACTGCGGAATCTGAATGGTC
Coll VI	GAACTTCCCTGCCAAACAGA	CACCTTGTGGAAGTTCTGCTC
CCN2	GAAGGGCAAAAAGTGCATCC	CAGTTGTAATGGCAGGCAC
TGF-ß1	TGGAGCAACATGTGGAACTC	GTCAGCAGCCGGTTACCA
TIMP-1	GCATCTGGCATCCTCTTGTT	CTCGTTGATTTCTGGGGAAC
CCN1	ATGAAGACAGCATTAAGGACTC	TGCAGAGGGTTGAAAAGAAC
CCN3	TGAAGTCTCTGACTCCAGCATT	TGGCTTTCAGGGATTTCTTG
m18S	CGGCTACCACATACCAAGGAA	GCTGGAATTAACCGCGGCT

### Tissue preparation and histological studies

One portion of liver tissue (left lobe) was fixed in 10% buffered formalin, processed, and then embedded in paraffin. Paraffin sections of 4 µm were stained with Picro Sirius Red (PSR) to evaluate total crosslinked collagen accumulation and stained by immunohistochemistry to detect collagen-I protein. Liver tissue (medial lobe) frozen at collection in OCT was sectioned at 6 µm thickness and stained by immunohistochemistry to detect F4/80 protein. The primary antibodies used were anti-collagen-I (1:400 dilution, AB765P, Millipore, Billerica, USA), and anti-F4/80 (1:500 dilution, ab6640, Abcam, Cambridge, UK). Appropriate biotin conjugated secondary antibodies were followed by Vectastain ABC kit (Vector Laboratories, Burlingame, CA). Collagen by Sirius Red Stain and Collagen-I, and F4/80 staining were scored for overall staining intensity. In brief, slides were examined under light microscopy visually as described. For the same scored measure of interest, all the sections were stained in the same batch and then scored in the same session. All sections were photographed using an Olympus Provis AX70 microscope (Olympus Optical Co., Japan). For each slide, 10 fields (which collectively cover the majority of the section), were randomly chosen for each sample scoring, at 200×magnification. The sections were viewed by two independent observers blinded to the group source of the sample section. Staining was scored as a value between 0 and 4, where 0 was no staining, and 4 was very intense staining, with reporting as an average of the two observers scores, as previously published (Lo et al. 2011).

### Preparation of tissue lysates for protein quantification and immunoblot analysis

Frozen liver tissue was homogenized in cold RIPA buffer containing Roche protease inhibitor cocktail and 50 mM NaF. The crude impurities were removed by centrifugation at 10,000 g for 20 min at 4 °C. Fat remaining on the tube wall was carefully removed in order to prevent contamination of the homogenate and supernatant was collected. Total protein concentration was measured using the DC Protein Assay (Bio-Rad Laboratories). Samples were loaded at 50 µg of total protein per lane and separated by a 4–12% gradient SDS-PAGE gel (Invitrogen). Proteins were electrotransferred onto nitrocellulose membrane and membranes were blocked with 5% skim milk/TBS with 0.1% (vol/vol) Tween 20 for 1 h, followed by incubation with each of anti-total-ERK (1:500 titre, 9102S, Cell Signaling, Danvers, MA, USA), anti-phospho-ERK (1:500, 9106S, Cell Signaling), anti-total-p38 (1:500, 9212, Cell Signaling), anti-phospho-p38 (1:500, 9211, Cell Signaling), anti-total-JNK (1:500, 9252, Cell Signaling), anti-phospho-JNK (1:500, 9251, Cell Signaling), anti-CCN1 (AF4055, R&D Systems, Minnesota, USA), or anti-CCN3 (1:500 titre, Cell Signaling) antibodies, each in TBST containing 1% skim milk overnight at 4 °C. After washing, the membranes were incubated with horseradish peroxidase-conjugated secondary antibody (1:5000) for 2 h at room temperature. The bands were visualized using enhanced chemiluminescence (Amersham Biosciences, Piscataway, NJ). In each case, ɑ-tubulin (1:5000, ab7291, Abcam) or GAPDH (1:5000 titre, Ab8245, Abcam) was used as a loading control. The intensity of the band in each case was quantitated by ImageLab software (Bio-rad, Hercules, CA). Final data of the band intensity for each group was calculated from at least three immunoblots performed as independent experiments.

### Oxidative stress measurement

Malondialdehyde (MDA) content was measured using the thiobarbituric acid reactive substances (TBARS) assay (Sigma-Aldrich, St. Louis, MO) by measuring absorbance value at a 532 nm wavelength. Superoxide-dismutase (SOD) activity was measured using the xanthine oxidase method to measure the absorbance value at 550 nm with the SOD kit (Sigma-Aldrich), according to the manufacturer’s instructions.

### Statistical analysis

**Fig. 2:**
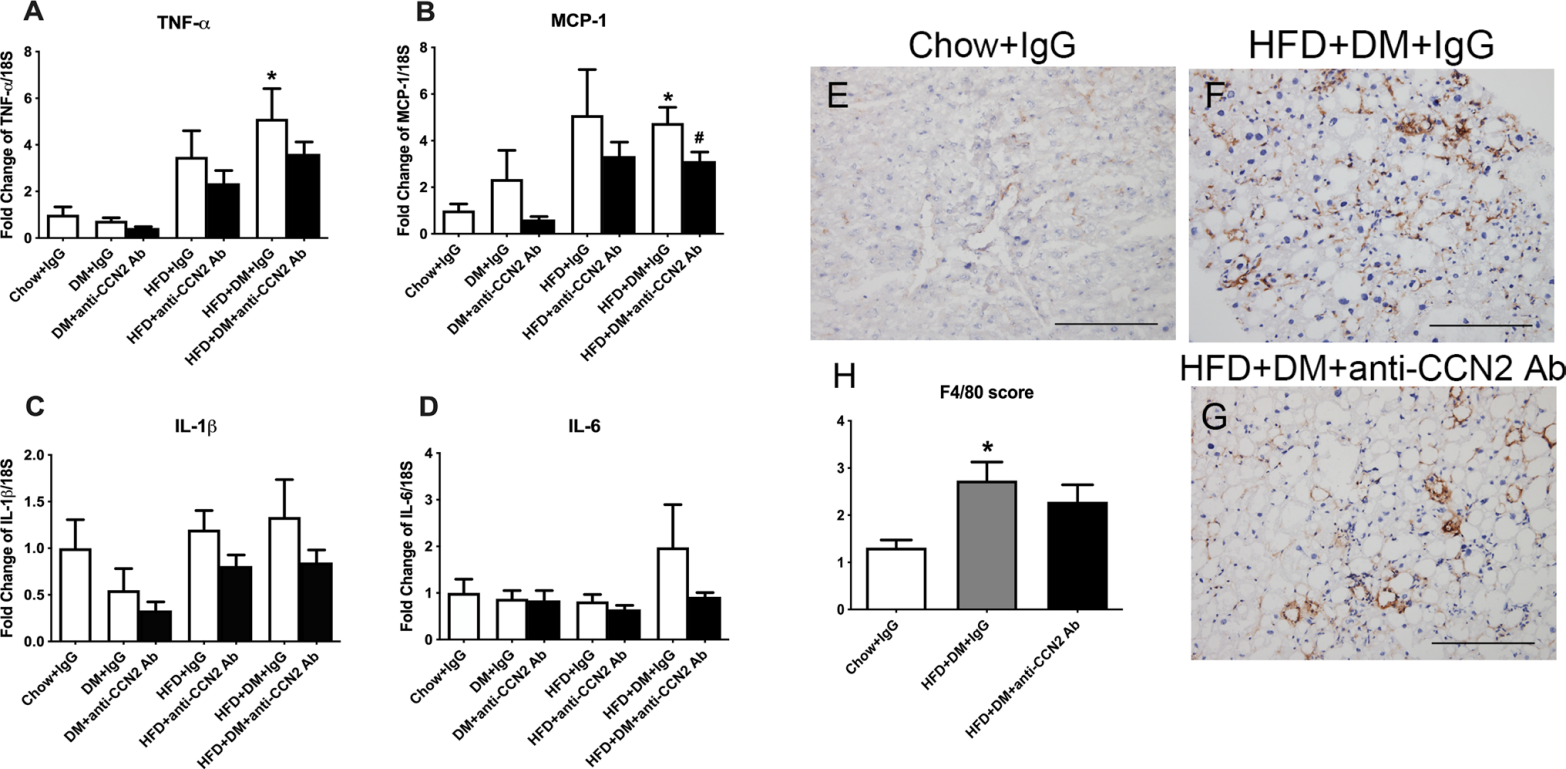
Gene expression of hepatic inflammation markers and F4/80 immunohistochemistry. TNF-α (**A**), MCP-1 (**B**), IL-1ß (**C**), IL-6 (**D**) mRNA quantitation. Data are shown as mean ± SEM as fold change of Chow + IgG. Representative images for F4/80 immunohistochemistry (**E**–**G**) are shown at 200 × magnification. Scale bar 200 µM. Data are mean ± SEM of staining intensity scores (**H**). *p < 0.05, significantly different from Chow + IgG. #*p* < 0.05, significantly different from HFD + DM + IgG

**Fig. 3:**
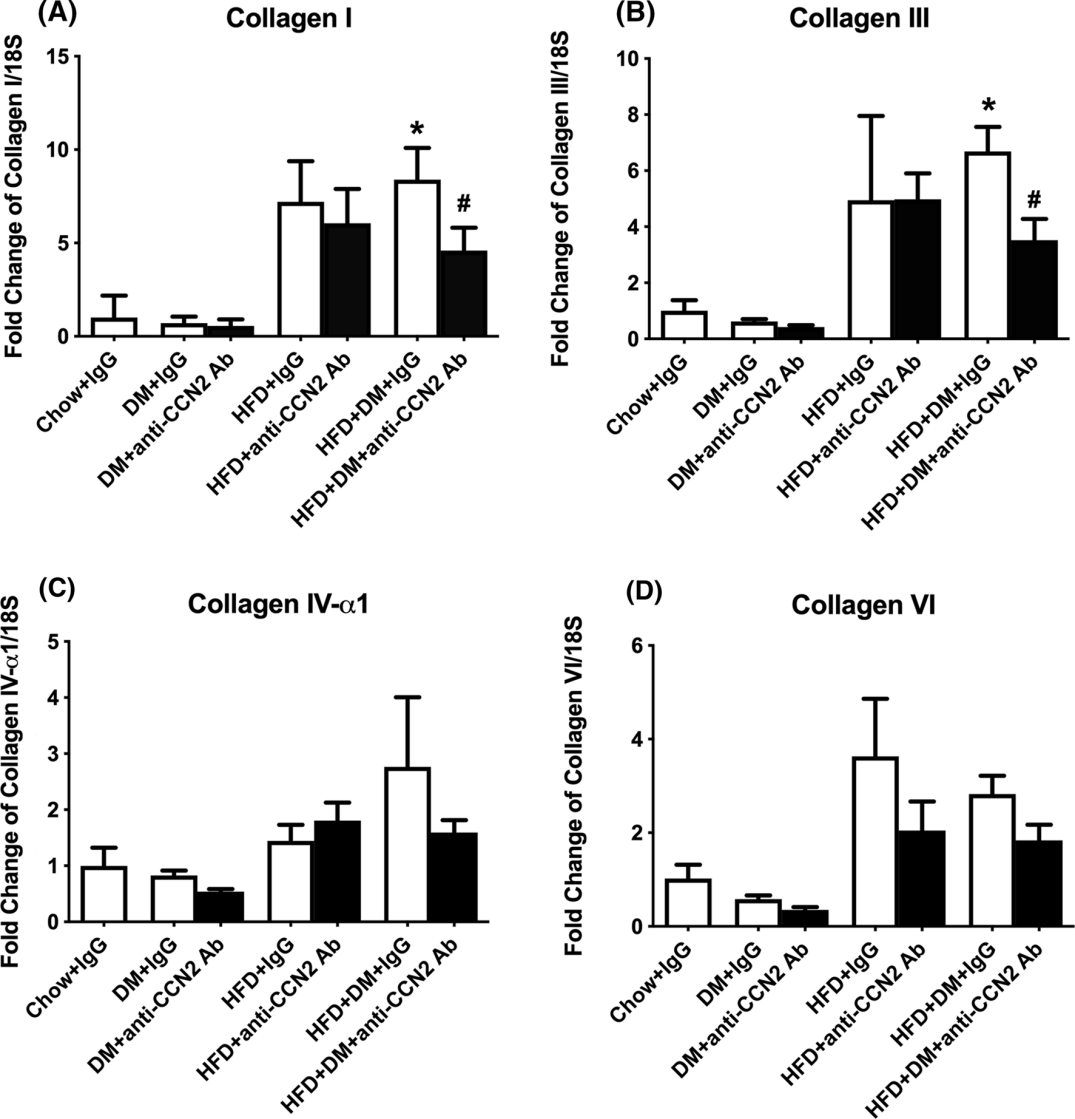
Gene expression of hepatic markers. Collagen-I (**A**), Collagen-III (**B**), Collagen-IV-ɑ1 (**C**), and Collagen-VI (**D**), mRNA quantitation. Data are mean ± SEM as fold change of Chow + IgG. *p < 0.05, significantly different from Chow + IgG; ^#^*p* < 0.05, significantly different from HFD + DM + IgG

**Fig. 4:**
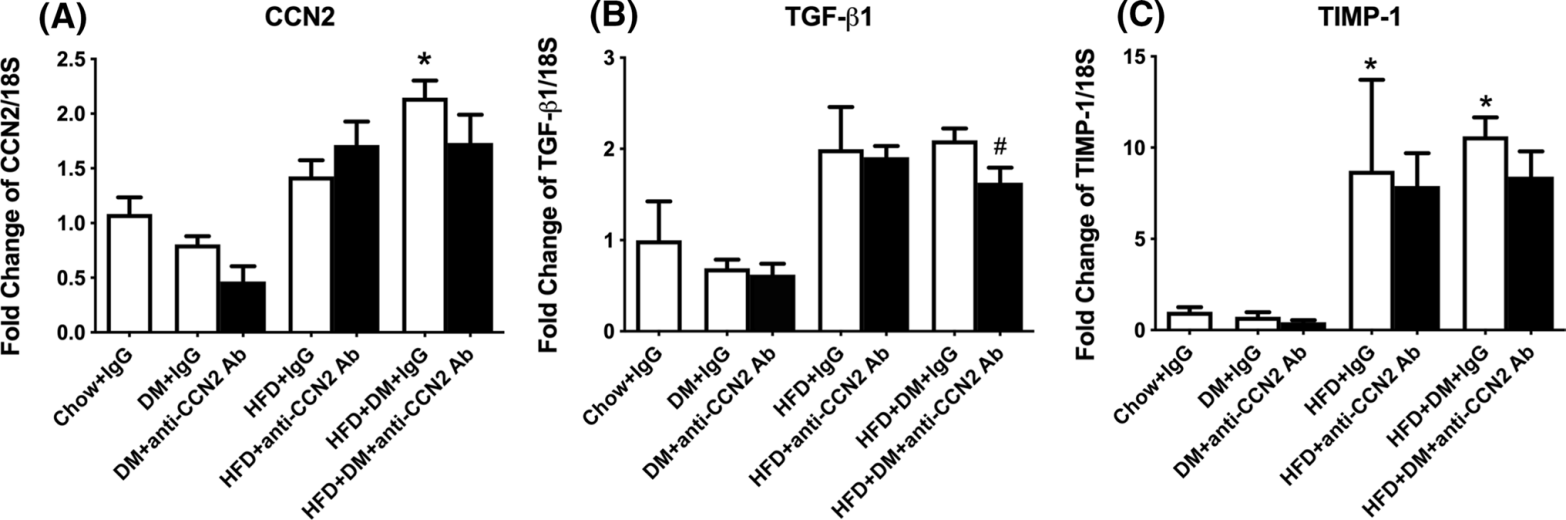
Gene expression of hepatic pro-fibrotic factors. CCN2 (**A**), TGF-ß1(**B**), and TIMP-1 (**C**) mRNA quantitation. Data are shown as mean ± SEM of fold change of Chow + IgG. *p < 0.05, significantly different from Chow + IgG; ^#^*p* < 0.05, significantly different from HFD + DM + IgG

**Fig. 5:**
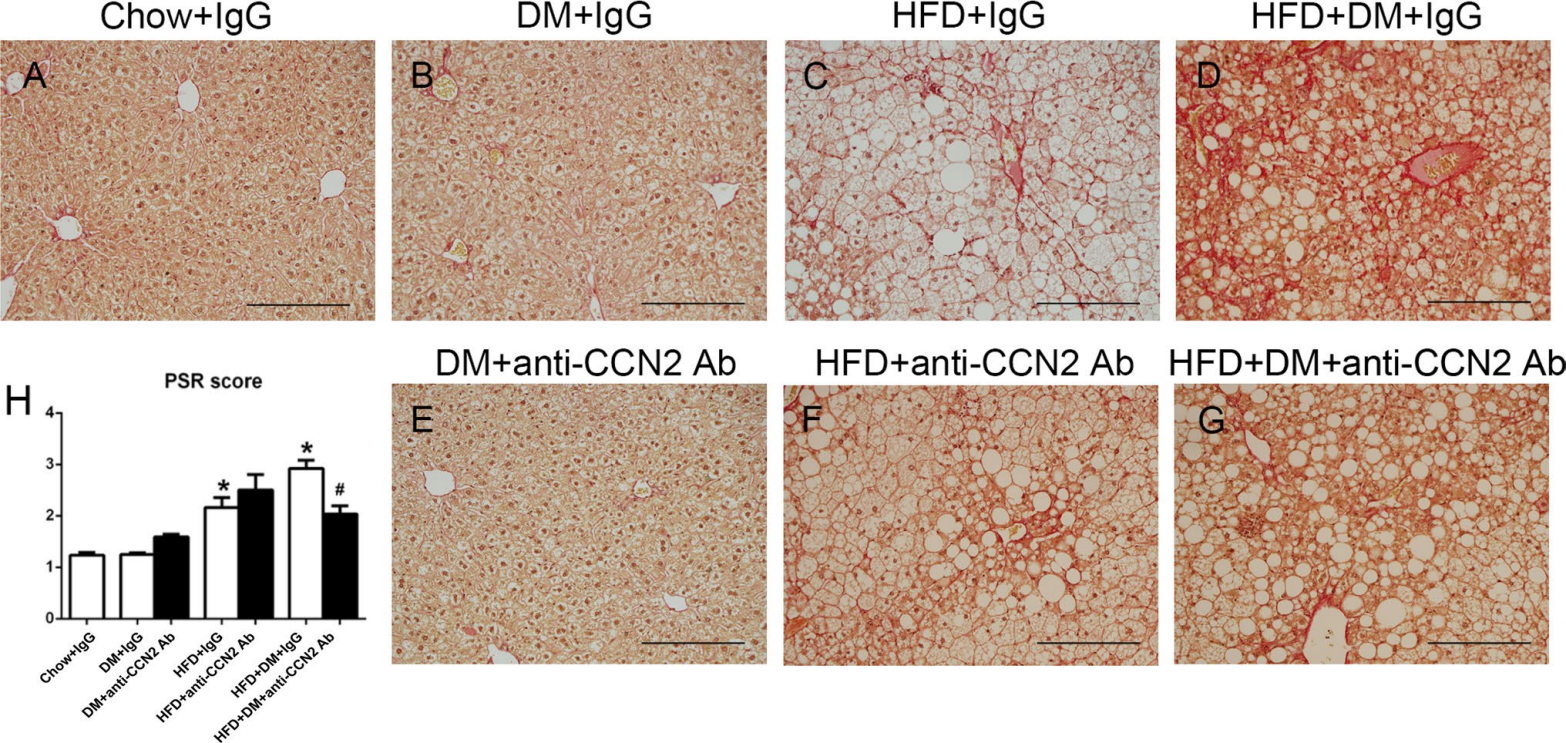
Liver Picro Sirius Red staining. Representative images of Picro Sirius Red staining (**A**–**G**) at 200 × magnification. Scale bar 200 µM. Data are mean ± SEM in staining amount and intensity scores (**H**). *p < 0.05, significantly different from Chow + IgG; ^#^*p* < 0.05, significantly different from HFD + DM + IgG

**Fig. 6:**
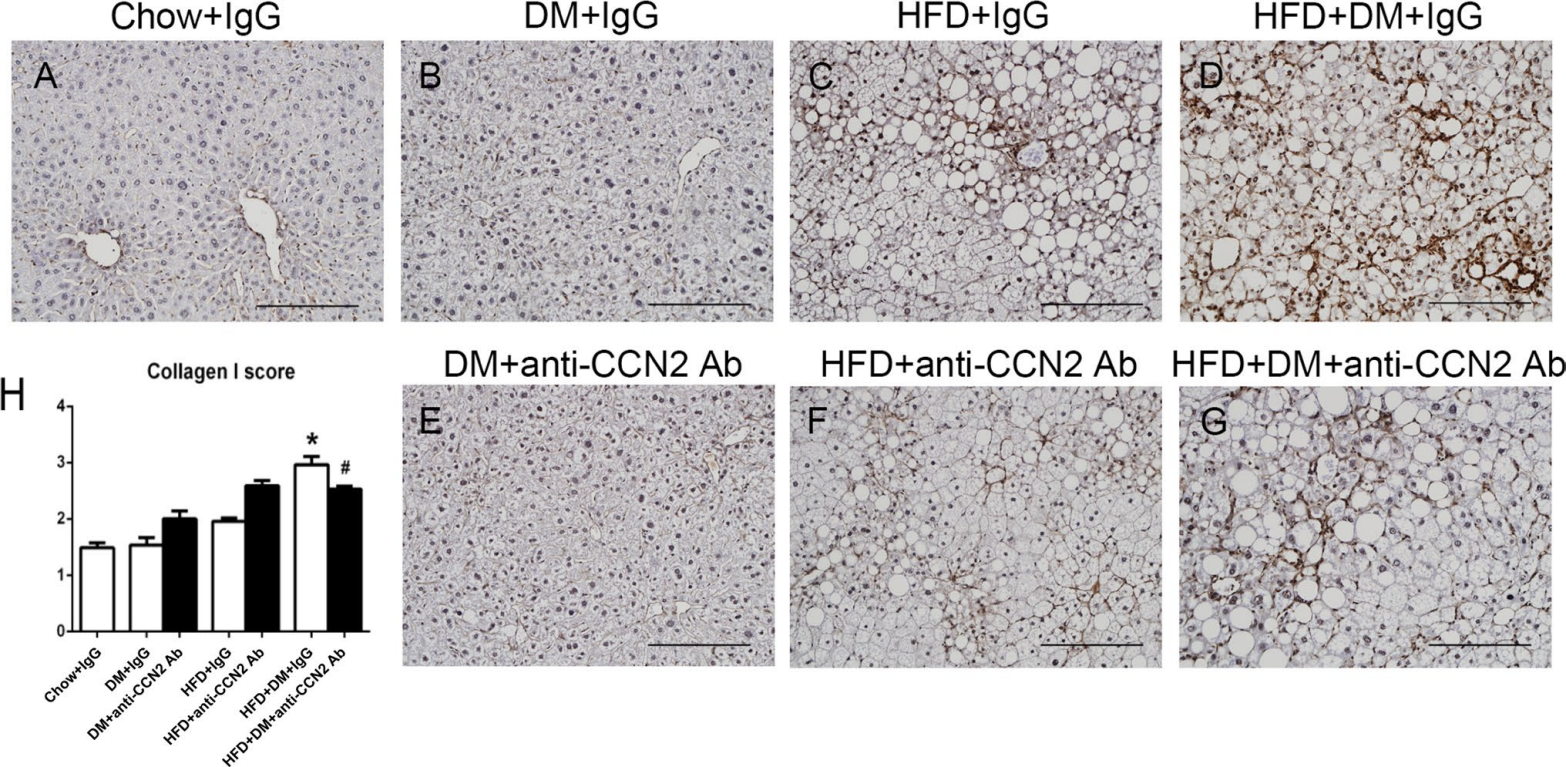
Liver type-I Collagen immunohistochemistry. Representative images for type-I Collagen immunohistochemistry (**A**–**G**) at 200 × magnification. Scale bar 200 µM. Data are mean ± SEM of staining amount and intensity scores (**H**). *p < 0.05, significantly different from Chow + IgG; ^#^*p* < 0.05, significantly different from HFD + DM + IgG

**Fig. 7:**
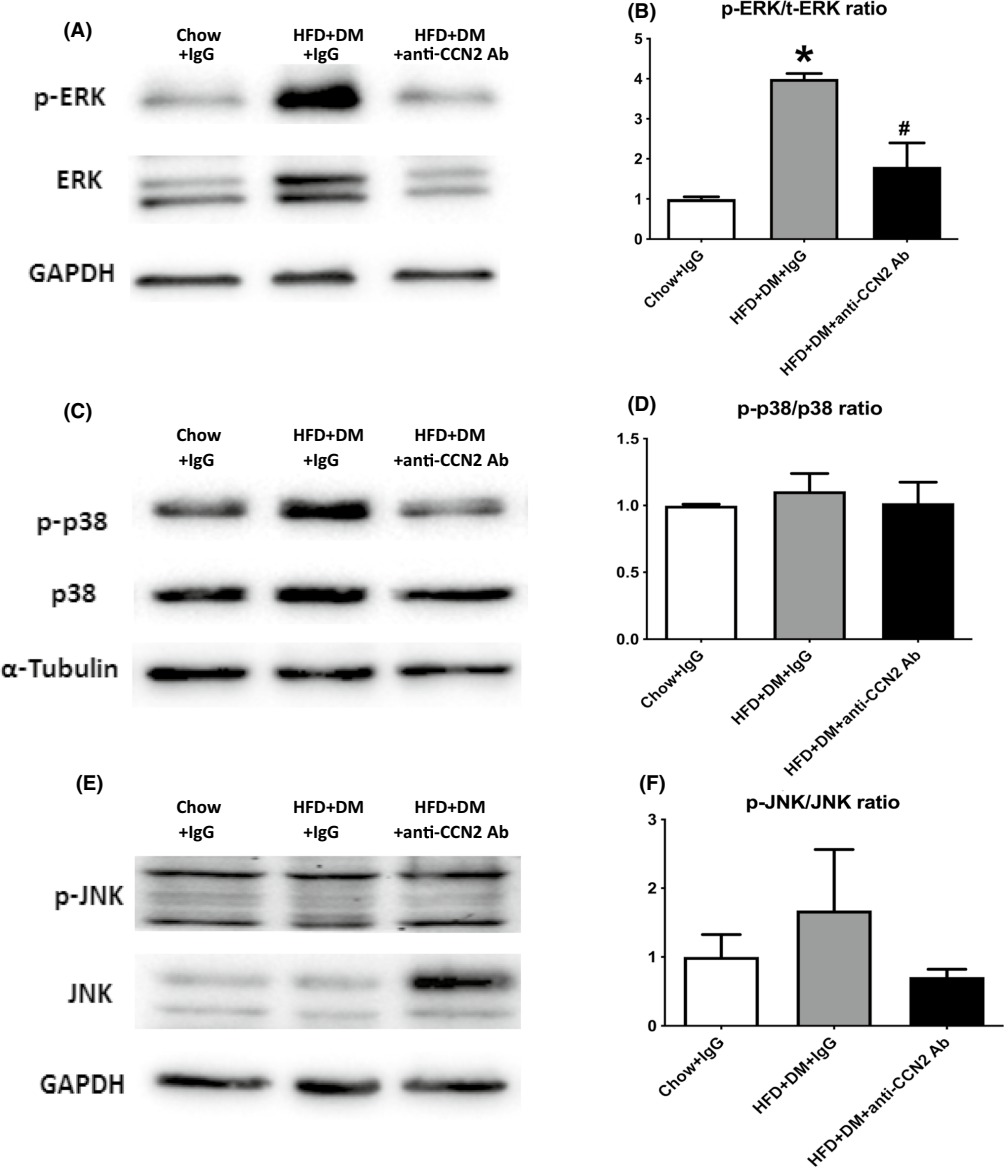
Effects of CCN2Ab on MAPK signaling pathway in high fat fed with diabetes mice. **A** detection of phospho-ERK, total ERK by immunoblot; GAPDH is a protein loading control; **C** detection of phospho-p38, total p38 by immunoblot; ɑ-tubulin is a protein loading control; **E**. detection of phospho-JNK, total JNK by immunoblot; GAPDH is a protein loading control. Band intensity in the immunoblot was semi-quantitated using ImageLab (**B**, **D** and **F**). Data is mean ± SEM as fold change of Chow + IgG. *p < 0.05, significantly different from Chow + IgG, ^#^*p* < 0.05, significantly different from HFD + DM + IgG

**Fig. 8:**
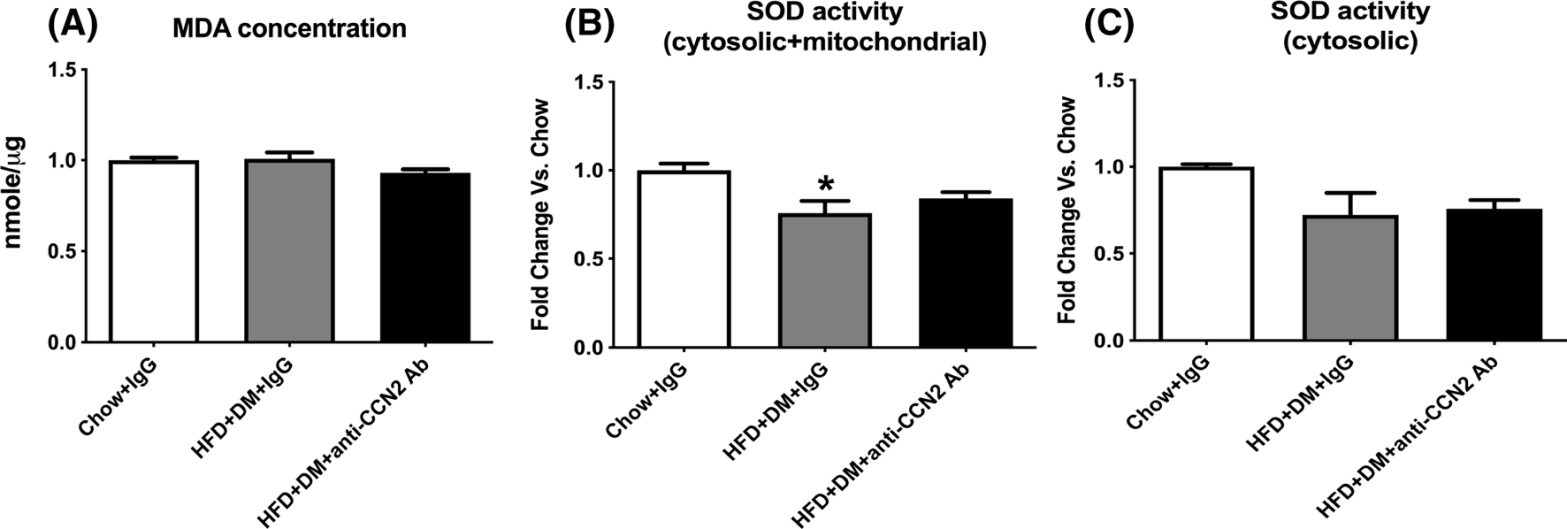
Effects of CCN2Ab on MDA content, and SOD activity in high fat fed with diabetes mice. MDA content (**A**), total SOD activity (**B**) and cytosolic SOD activity (**C**) were measured in high fat feeding with diabetes mice. Data is shown as MDA concentration in Fig. **A** and as mean ± SEM as fold change of Chow in Fig. **B** and **C**. *p < 0.05, significantly different from Chow, by un-paired *T*-test

**Fig. 9:**
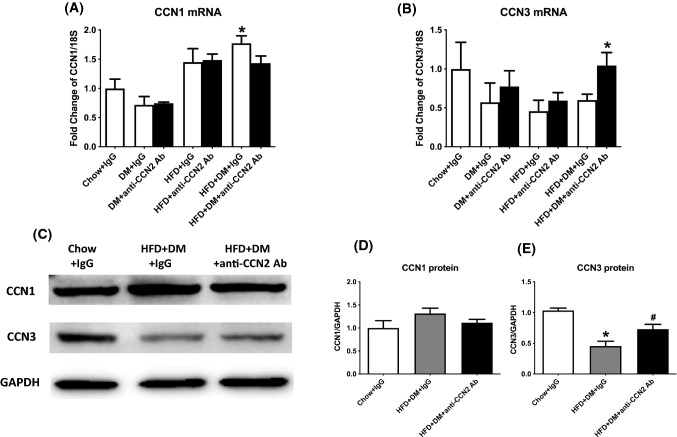
Effects of CCN2Ab on CCN1 and CCN3 expression in high fat fed with diabetes mice. CCN1 (**A**), and CCN3 (**B**) mRNA quantitation. Detection of CCN1 and CCN3 by immunoblot; GAPDH was a protein loading control (**C**). Band intensity in the immunoblot was quantitated using ImageLab (**D** and **E**). Data is shown as Mean ± SEM as fold change of Chow + IgG. *p < 0.05, significantly different from Chow + IgG, ^#^*p* < 0.05, significantly different from HFD + DM + IgG

Results are shown as mean ± standard deviation (SD) in Table 2 and as mean ± standard error of the mean (SEM) in Figs. 2, 3, 4, 5, 6, 7, 8 and 9 inclusive. All data were compared using one-way ANOVA followed by post hoc analysis using Bonferroni’s multiple comparisons test or an un-paired T-test. Statistical significance was accepted at *p* < 0.05.

**Table 2:** Animal characteristics and metabolic findings

	Body weight (g)	Blood glucose (mmol/L)	ITT (glucose AUC, % change cf CHOW)
Chow + IgG (n = 5)	33.12 ± 2.20	8.9 ± 1.0	100.0 ± 23.9
DM + IgG (n = 5)	30.14 ± 1.69	18.0 ± 4.8*	240.8 ± 91.5*
DM + anti-CCN2 Ab (n = 5)	27.28 ± 7.04	19.2 ± 5.8*	232.0 ± 53.2*
HFD + IgG (n = 5)	51.48 ± 8.24*	9.2 ± 1.1	124.1 ± 21.0
HFD + anti-CCN2 Ab (n = 5)	52.86 ± 1.25*	6.7 ± 0.5	118.6 ± 15.7
HFD + DM + IgG (n = 10)	43.55 ± 7.20*	14.1 ± 3.3*	140.0 ± 28.0
HFD + DM + anti-CCN2 Ab (n = 10)	42.24 ± 7.34*	14.3 ± 5.3*	152.1 ± 32.0

Data are mean ± SD, at 25 weeks of standard chow or high fat feeding including the final 10 weeks diabetes with or without treatment

*p < 0.05 versus Chow + IgG alone

## Results

### Effects of CCN2Ab on metabolic measures

Body weight of HFD+IgG and HFDDM+IgG mice at week 25, as expected, was greater than Chow mice. In the Chow-diabetes group and the HFD plus diabetes group, as expected, blood glucose levels were elevated in each and they were also insulin resistant as shown with a greater glucose area under the curve (AUC) value than Chow fed mice when subjected to an ITT (Table 2). Notably, none of these metabolic measures were affected by the CCN2Ab compared with the control IgG in each of the respective mouse groups (Table 2).

### Effects of CCN2Ab on hepatic inflammatory markers

While it is mainly thought to be pro-fibrotic, CCN2 has also been reported to induce an inflammatory response (Abraham 2008; Sanchez-Lopez et al. 2009). To assess if inflammatory changes are affected by the anti-CCN2 neutralizing antibody, expression of key hepatic pro-inflammatory cytokines were examined at the mRNA level in liver tissue including TNF-ɑ, MCP-1, IL-1β and IL-6. High-fat feeding induced TNF-ɑ mRNA ~ 3.5 fold compared with Chow + IgG and the induction was inhibited by CCN2Ab in HFD mice. TNF-ɑ mRNA was also significantly elevated ~ 5.1 fold in the HFD + DM + IgG group compared with Chow + IgG and this induction was normalized by CCN2Ab (Fig. 2A). Intrahepatic MCP-1 mRNA expression was significantly increased in the HFD plus diabetes group by 4.7fold compared with Chow + IgG, and the CCN2Ab treatment inhibited MCP-1 mRNA induction in this group (Fig. 2B). For IL-1β (Fig. 2C) and also IL-6 (Fig. 2D), there was no changes observed in any group. Mouse macrophage marker F4/80 was assessed by immunohistochemistry (Fig. 2E, G). F4/80 immunopositivity was elevated in HFD+DM+IgG compared with Chow+IgG (*p* < 0.05; Fig. 2H) and appeared to be partially reversed by the CCN2Ab, but not to statistical significance. In contrast to effects of CCN2 Ab treatment observed on some inflammatory marker mRNA levels in liver, by H&E staining, no effect of the CCN2 Ab treatment on hepatic steatosis compared with control IgG treatment was observed in any mouse group examined (neither HFD, nor in HFD+DM, data not shown).

### Effects of CCN2Ab on intrahepatic fibrosis in the high fat feeding with diabetes mouse model

CCN2 as an upstream and downstream mediator of TGF-β bioactivity, can stimulate the expression of many ECM proteins, and also decrease matrix degradation by matrix metalloproteinase through the upregulation of tissue inhibitor of metalloproteinase (TIMP-1) in hepatic stellate cells and myofibroblasts (MFB) (McLennan et al. 2004). Dysregulation of CCN2 is involved in several diseases which involve fibrosis (Chen et al. 2009; Panek 2009; Rachfal and Brigstock 2003). In this study, in order to investigate the effects of targeting CCN2, the impact on ECM was determined by RT-qPCR analysis of mRNA expression of collagen-I, -III, -IV, -VI, CCN2 itself, TGF-β, and TIMP-1.

Expression of ECM components is shown in Fig. 3. Collagen-I mRNA expression was significantly increased in the HFD+DM+IgG group ~ 8.4 fold, compared with Chow+IgG and was significantly prevented by CCN2Ab (Fig. 3A). Similarly for collagen-III mRNA levels, HFD+IgG animals showed a non-significant fivefold induction compared with Chow-fed+IgG animals, while this induction was significant in the HFD+DM+IgG group, at 6.7- fold greater than Chow+IgG. The induction of collagen-III mRNA in the HFD+DM+IgG group was significantly prevented by the CCN2Ab (Fig. 3B). High-fat feeding and high-fat feeding with diabetes increased the mRNA levels of both collagen-IV and collagen-VI only as trends, without showing statistical significance. The CCN2Ab showed a trend towards lowering the abundance of these collagens in the HFD + DM group (Fig. 3C, D).

Expression of a series of growth factors and effects of CCN2Ab treatment are shown in Fig. 3. The CCN2 mRNA level was significantly induced in the HFD + DM + IgG group by ~ 2.1-fold compared to Chow + IgG, which was partially inhibited by the CCN2Ab treatment (Fig. 4A). TGF-β mRNA levels showed a non-significant trend towards elevation in the HFD + IgG and HFD + DM + IgG groups, and the CCN2Ab treatment significantly inhibited the mRNA expression induced in the HFD + DM + IgG group (Fig. 4B). TIMP-1 mRNA levels were elevated in both HFD + IgG and HFD + DM + IgG groups, to 8.7-fold and 10.6-fold respectively, compared with Chow + IgG, but, notably, these changes were not affected by the CCN2Ab treatment (Fig. 4C).

In order to investigate possible effects of neutralizing CCN2Ab on the protein levels of fibrotic markers in the NASH model, PSR staining was used to assess total crosslinked collagen accumulation and Collagen-I protein levels was examined by immunostain. Figure 5 shows representative images of PSR staining in the liver sections from each group of mice. As expected, and as previously observed by our group in this model (Lo et al. 2011), the diabetes alone group did not show differences compared with Chow-fed animals (Fig. 5B). The PSR staining score of the HFD + IgG group was ~ twofold induced compared with Chow + IgG, and it was not affected by the CCN2Ab antibody (Fig. 5C, F and H). In contrast, the combination of HFD + DM + IgG showed a statistically significant induction in the collagen PSR stain ~ 2.5-fold within the liver lobule sited around the central veins and portal tract as well as across the liver parenchyma, and which was significantly lowered by CCN2Ab therapy (Fig. 5D, G and H). Type-I Collagen is the main collagen induced in NASH and was found to be the most induced in the HFD + DM + IgG group and that induction was statistically significantly inhibited by CCN2Ab only in that group (Fig. 6D, G and H).

### Potential mechanisms of NASH prevention by CCN2Ab

CCN2 activates the MAPK cascade in several cell types (Crean et al. 2002; Wahab et al. 2005; Yosimichi et al. 2006), and in other studies some of the CCN2 bioactivity is reported to be mediated by activation of the MAPK signaling pathway (Nagai et al. 2009). To investigate signaling pathways through which CCN2 may contribute to NASH progression, the intrahepatic levels of ERK1/2, p38 and JNK MAPK proteins in HFD + DM mice, with or without CCN2Ab treatment in vivo, were measured by immunoblotting. HFD + DM was the focus of these analyses because the combined metabolic insult causes the greatest induction of fibrosis in the NASH model.

The phosphorylation of ERK in liver lysate was found to be significantly induced in the HFD + DM mouse model compared with normal chow fed mice, and the phosphorylation of ERK was lower following in vivo CCN2Ab treatment (Fig. 7A and B). In contrast to the findings for ERK phosphorylation, no significant difference in phosphorylation of p38 was found in HFD + DM mice, nor in the antibody treatment group, compared to normal chow fed mice (Fig. 7C and D). Total JNK and phospho-JNK were each not significantly changed in the HFD + DM model compared to Chow + IgG. While total JNK was significantly induced in the CCN2Ab treatment group (quantitation not shown), the phospho-JNK to total JNK ratio was not significantly affected by CCN2Ab treatment (Fig. 7E and F).

### Effects of CCN2Ab treatment on intrahepatic oxidative stress in the high fat feeding with diabetes mice

Oxidative stress is implicated in the pathogenesis of NAFLD (Sumida et al. 2013). Reactive oxygen species and lipid peroxidation products have been demonstrated to precede necro-inflammatory changes, hepatic stellate cell activation and collagen deposition (Sanchez-Valle et al. 2012). Antioxidant treatment, which decreases oxidative stress, also attenuates the severity of fibrosis in experimental steatohepatitis (Al-Busafi 2012; Vizzutti et al. 2010). As a potentially important pro-fibrotic molecule in hepatic fibrosis, CCN2 is upregulated by oxidative stress in various diseases (Aragno et al. 2008; Elmarakby and Sullivan 2012). However, whether blocking CCN2 activity may ameliorate oxidative stress in NASH has not been clarified.

In our study, we detected oxidative stress by measuring the lipid peroxidation level and by determining antioxidant enzyme activity. MDA concentration, which reflects oxidative stress, compared with the Chow group, was not changed in the HFD + DM group, and was slightly decreased by the CCN2Ab treatment (Fig. 8A). Antioxidant total SOD activity (mitochondrial and cytosolic SOD combined) was significantly decreased in the HFD + DM + IgG group compared with Chow, and trended towards an increase in the CCN2Ab treatment group (Fig. 8B). Cytosolic SOD trended towards decrease in the HFD + DM group and was not affected by the CCN2Ab treatment (Fig. 8C).

### Regulation of CCN family member by CCN2Ab treatment in the high fat feeding with type 2 diabetes model

Across CCN family members, CCN protein expression or activities are dysregulated in various pathological conditions, including inflammation and fibrosis (Leask and Abraham 2006). The CCNs have important roles as matricellular proteins, involved in the connection between cell surface and extracellular matrix (ECM) structure and signaling (Leask and Abraham 2006). CCN1 (Kim et al. 2013), CCN2 (Rachfal and Brigstock 2003), and CCN3 (Borkham-Kamphorst et al. 2012) have each been studied to some degree in liver fibrosis. However, the changing profiles in response to blockade of CCN2 bioactivity and possible roles of other CCN family members in NASH development have not been clarified to date. In this study, the respective profile of CCN family member expression in experimental NASH was examined.

As a regulator expressed in myofibroblasts preventing fibrosis through induction of cellular senescence (Borkham-Kamphorst et al. 2014), CCN1 mRNA was unchanged in the CHOW fed diabetes alone group, whereas it was induced in the HFD alone model. CCN1 was also significantly induced in the HFD with added type 2 diabetes model, by ~ 1.7-fold compared with Chow. The CCN2Ab did not have effects in the diabetes nor in HFD alone groups, and it showed only a trend towards lowering CCN1 in the HFD + DM model (Fig. 9A). In addition, CCN1 protein level was induced in the HFD + DM model compared with Chow to ~ 1.3-fold, and was unaffected by CCN2Ab (Fig. 9C and D). As an anti-fibrotic molecule in the CCN family, the gene expression of CCN3 showed a trend to being lower in the diabetes alone, HFD alone, and the HFD with diabetes group, each compared with the Chow group. Blocking CCN2 by CCN2Ab showed a trend only to normalisation of the CCN3 gene expression in diabetes alone and HFD alone groups, whereas CCN3 was clearly statistically significantly induced in the HFD + DM group (Fig. 9B). The CCN3 protein level was significantly decreased in the HFD + DM model compared with Chow and the CCN2Ab partially normalized the CCN3 protein level observed in this model (Fig. 9C and E). The expression levels of CCN4, CCN5 and CCN6 were not significantly changed across DM, HFD or HFD + DM groups, and were not affected by CCN2Ab in any mouse group (data not shown).

## Discussion

Intervention with anti-CCN2 neutralizing antibody in two NAFLD models was studied in this work. Data demonstrated that targeting CCN2 bioactivity by the CCN2Ab for 10 weeks provided some protective effects against NASH induced by high fat feeding with diabetes, particularly in inflammation markers and in fibrosis end-points. The main aim in this study was to investigate the role of CCN2 in NASH development and to examine whether its inhibition may be a potential target as a preventive therapy for NASH.

Some insights into mechanism of action of the CCN2Ab in this NASH model were realised. If there is a central mechanism it is possibly through affecting known pro-inflammatory and/or pro-fibrotic effects of CCN2. Studies have suggested a potential role for CCN2 in chronic inflammatory diseases, such as atherosclerosis, rheumatoid arthritis, inflammatory kidney disease and neuroinflammatory pathologies (Kular et al. 2011). Leukocyte, especially macrophage, recruitment is an important event involved in inflammation (Muller 2013) and CCN2 can contribute to this phenomenon (Sanchez-Lopez et al. 2009). CCN2 was shown to induce adhesion of peripheral blood activated monocytes and macrophages in vitro, and to promote infiltration of T lymphocytes and monocytes in the renal interstitium in vivo (Sanchez-Lopez et al. 2009; Kular et al. 2011). CCN2 also functions by regulating cytokine and chemokine expression in various cell systems. In cardiomyocytes, CCN2 increases the expression of the pro-inflammatory cytokines TNFα, IL-6, MCP-1 and IL-8 (Wang et al. 2010). To date, the role of CCN2 in the inflammation of NASH is unclear. The current work showed that blocking the bioactivity of CCN2 decreased MCP-1 gene expression and it improved TNF-α levels in the HFD + DM NASH model, suggesting that CCN2 is pro-inflammatory in this model. Related signalling pathways including NF-ĸB need to be investigated in future studies.

In addition to its pro-inflammatory role, CCN2 is regarded as an important mediator involved in fibrotic processes; it stimulates fibroblast growth and ECM protein up-regulation in vitro and in vivo. Over-production of CCN2 has been proposed to be important in pathways leading to fibrosis in many tissues including skin, kidney, heart and lung (Leask and Abraham 2004). However, whether CCN2 contributes to fibrosis progression in NASH combined with type 2 diabetes has been unclear. The data in this research shows that blocking CCN2 bioactivity with neutralizing antibody attenuated fibrotic changes, including inhibition of collagen and pro-fibrotic marker gene expression induction and also key ECM and profibrotic protein levels, in the liver. Our laboratory has previously reported that CCN2 may contribute to matrix accumulation not only through ECM formation but also through the inhibition of matrix degradation via TIMP-1 induction by CCN2 (McLennan et al. 2004). In this study, in contrast to robust effects of CCN2 bioactivity in human renal mesangial cells (McLennan et al. 2004), TIMP-1 gene expression was only minimally normalized by CCN2Ab treatment in the HFD + DM NASH model. Nonetheless, the results in this study indicate that CCN2 plays an important pro-fibrotic role during NASH fibrosis progression, especially in the HFD + DM model.

The MAPK family of serine-threonine protein kinases can be activated by various and multiple extracellular stimuli (Wetzker and Bohmer 2003). Three major MAPK subfamilies, including ERK, p38 and JNK, mediate a number of downstream events such as cellular proliferation, differentiation, and apoptosis by activating transcription factors, in a specific cell type and situation dependent manner (Wetzker and Bohmer 2003; Kyriakis and Avruch 2001). CCN2 has been shown to activate MAPK cascades in several cell types (Crean et al. 2002; Wahab et al. 2005; Yosimichi et al. 2006). We examined whether any of these pathways were involved in matrix regulation in the NASH mouse model by inhibiting CCN2 with the CCN2Ab. The result showed that blocking CCN2 caused lowered phosphorylation levels of ERK. This suggests that CCN2Ab may attenuate liver fibrosis through inhibiting the ERK signalling pathway. The increased level of total JNK in the CCN2Ab treatment group may be due to inhibition of one pathway causing the induction of another pathway, including total levels of protein.

Oxidative stress has been implicated in the pathogenesis of NAFLD (Sumida et al. 2013). The role of oxidative stress in steatosis and its progression to steatohepatitis has been widely discussed. Increased levels of reactive oxygen species (ROS) and lipid peroxidation products (such as MDA) and decreased level of antioxidant enzymes (such as SOD and catalase) and compounds (such as glutathione) have been reported in patients with NASH compared to healthy controls (Videla et al. 2004). In addition, oxidative stress can regulate CCN2 expression in different cell types (Elmarakby and Sullivan 2012; Videla et al. 2004). The data in this study does not clearly address whether CCN2 inhibition down-regulates oxidative stress, as overall, detectable changes in oxidative stress measures were small, and the trends towards normalization for the MDA level and SOD activity in the anti-CCN2 neutralizing antibody treatment group were not statistically significant.

In addition to CCN2, emerging studies have reported that the CCN protein family may be a relatively new appreciated class of mediators in inflammation and fibrosis processes (Kular et al. 2011). CCN1 is an important regulator of inflammation and wound repair, commonly being expressed in myofibroblasts of granulation tissue and it regulates fibrosis through cellular senescence (Lau 2011). CCN1 induces the expression of cytokines (TNF-α, IL-1β, IL-6 and IL-12b) and chemokines (MCP3) in murine macrophages, thus helping macrophages to participate as potent inducers and effectors in the Th1 response (Bai et al. 2010). CCN1 also induces the release of multiple growth factors and chemokines in cardiovascular inflammatory process (Grote et al. 2007). CCN3 counteracts CCN2 effects in several cell systems (Kawaki et al. 2008; Riser et al. 2009). It has been demonstrated that CCN3 alone, or in concert with other signals, can regulate neuro-inflammatory responses by exerting both pro- and anti- inflammatory actions (Dreau et al. 2010). Moreover, in a previous study, the overexpression of the CCN3 gene in renal fibroblasts caused a reduction in CCN2 production and the related fibrotic response (Riser et al. 2009). The anti-fibrotic action of CCN3 may be partly through direct inhibition of collagen induction, as well as inhibition of CCN2 mRNA induction, plus by CCN3 directly binding CCN2 protein and preventing CCN2 bioactivity (Abd El Kader, et al. 2013). However, the roles of other CCN family members involved in NASH progression remain unclear. In our study, CCN1 was upregulated in the HFD + DM NASH model suggesting that CCN1 may be a mediator promoting NASH progression, or as a counter-regulatory anti-fibrotic factor. Anti-fibrotic CCN3 was found to be downregulated at the gene and protein level in the HFD + DM model but was induced by CCN2Ab treatment. This indicates that CCN3 levels are inhibited in this pathological state, and inhibiting CCN2 effects can normalise its levels and perhaps the overall CCN3 bioactivity. In contrast, dysregulation of CCN4, CCN5 or CCN6 was not observed in the NASH model, suggesting that they do not play a major role in this model or in NASH fibrosis generally. Thus, in addition to CCN2, CCN1 and CCN3 may be novel therapeutic targets for NASH combined with type 2 diabetes with fibrosis. The precise mechanisms of action of CCN members in NASH progression, alone and in concert, remain to be investigated.

It is notable in this model that the use of the anti-CCN2 antibody prevented about half of the fibrosis induced in the model compared with chow fed mice. It could thus be concluded that CCN2 mediates only part of the fibrosis in the HFD + DM model. The antibody was added at the time diabetes was induced, which is at a time after 15 weeks of the initial high fat feeding and from that perspective it could be considered a form of delayed treatment rather than entirely a prevention regimen. The aim of the current work was to attempt to prevent the induction of NASH with fibrosis caused by adding diabetes to high fat feeding. In general, this did occur—the fibrotic response at the levels of both mRNA expression and collagen stains showed that the antibody prevented any fibrosis induction over and above that seen by HFD alone. Whether a regimen of commencing the CCN2Ab therapy at week 1 of the protocol before high fat feeding, or whether a higher dose or more intense regimen of CCN2Ab may have produced even greater prevention of fibrosis change in NASH is unknown, and was not able to be addressed in this work owing to limiting amounts of polyclonal antibody reagent for in vivo study, which even in the current regimen required 170 mg in total across all mice.

As the CCN2Ab therapy involved a systemic administration of an agent to inhibit CCN2 bioactivity, this suggests that systemic or other organ effects of CCN2 might have been responsible for protective effects observed in the liver. We have previously reported that CCN2 inhibits murine cell adipocyte differentiation in vitro, inducing a cellular profile found in insulin resistance (Tan et al. 2008). Others have found in humans (Yoshino et al. 2019), as we have in mice, that adipose tissue expression of CCN2 correlates with systemic insulin resistance. Whilst it is possible that systemic CCN2 may have mediated the NASH fibrosis in the current work, it is notable that the body weight, blood glucose levels and insulin sensitivity of the mice within each group (HFD, DM, and HFD + DM), were not affected by the CCN2Ab compared with the control IgG (Table 2). This whole body data thus suggests that the anti-CCN2 antibody was not having secondary effects on the liver through systemic glucoregulation and related metabolism, but was directly inhibiting liver pathology, especially in its hepatic anti-fibrotic effect, including in the profile of CCN2Ab preventing ERK-phosphorylation in the HFD + DM mice.

We examined only male mice in the current study as our published NASH fibrosis model induced by was generated in male mice (Lo et al. 2011) and others have preferentially utilised male mice for nutritional models (Rossmeisl et al. 2003) and in CCN liver fibrosis dysregulation research (Kim et al. 2013). Future studies could explore development of a NASH fibrosis model in female mice, including potential effects of estrus, and liver CCN targeting. In addition, blood liver enzymes were not examined as we (Lo et al. 2011) and others (Kim et al. 2013), have not previously found strong associations of these murine measures with histological liver inflammation or fibrosis. Despite these inherent limitations of the CCN2 bioactivity regimen used in a single time course and concentration protocol, the collective data herein indicate that inhibition of CCN2 bioactivity prevents induction of fibrosis in the NASH mouse model that was studied, implicating CCN2 as a mediator in human NASH induced by type 2 diabetes. Future preclinical studies targeting CCN2 expression specifically in the liver and its cell types could complement the studies described in this current work.
